# Evaluation of micronutrient and nutritional status among preschool children in Jordan: results from a Nationwide survey

**DOI:** 10.3389/fnut.2024.1423904

**Published:** 2024-07-24

**Authors:** Rawhieh Barham, Reema Tayyem, Lama Al-Majali, Buthayna Al-Khatib, Ayoub Al Jawaldeh

**Affiliations:** ^1^Nutrition Department, Ministry of Health, Amman, Jordan; ^2^Department of Human Nutrition, College of Health Sciences, Qatar University, Doha, Qatar; ^3^WFP, World Food Programme, Amman, Jordan; ^4^UNICEF, United Nations International Children’s Emergency Fund, Amman, Jordan; ^5^Regional Office for the Eastern Mediterranean, World Health Organization, Cairo, Egypt

**Keywords:** micronutrients deficiency, nutritional status, preschool children, feeding indicators, national survey

## Abstract

**Background:**

Jordan faces various malnutrition challenges, including undernutrition, micronutrient deficiencies, overweight, obesity, and diet-related non-communicable diseases. The country has shifted from issues of undernutrition to rising rates of overweight and obesity, while still dealing with micronutrient deficiencies. The 2010 national survey revealed high rates of iron and vitamin D deficiencies among preschool children, with about 20% experiencing vitamin A deficiencies. The goals of the 2019 Jordan National Micronutrient & Nutrition Survey (JNMNS) include assessing feeding practices of infants and young children, determining the frequency of consuming micronutrient-rich foods, evaluating causes of anemia, assessing the health status of specific subgroups, and comparing findings to the 2010 survey.

**Methods:**

JNMNS 2019 was a comprehensive national cross-sectional survey structured across four strata. Within each stratum, 40 primary sampling units were chosen in proportion to their size based on the 2015 Jordan census. Subsequently, the Department of Statistics conducted household listings in each PSU. Separate response rates were assumed for households and target groups, encompassing interviews, anthropometric measurements, and specimen collection. The survey aimed to collect data from 2,210 households, including interviews and anthropometry for 1,232 preschool children, with blood samples obtained from 992 of them.

**Results:**

The findings revealed no severe anemia cases, but 11% of preschoolers aged 12–59 months were anemic. Iron deficiency affected 22.4%, with 5% having iron deficiency anemia. Vitamin D deficiency increased to 22.9% in 2019. Stunting and wasting rates improved slightly to 6.3 and 0.1%, respectively. Overweight and obesity rates remained stable at 6.2 and 2.1%. Anemia decreased by 5–6%, but iron deficiency rose by 7%. Vitamin A deficiency decreased, but iron deficiency anemia remained largely unchanged. Undernutrition was rare, but vitamin D deficiency affected 27.7% of preschoolers, impacting growth and immunity. Iron deficiency, affecting 25% of children, poses a risk to cognitive development. Overweight or obesity affected 9% of children, a medium public health issue according to the WHO. While malnutrition rates are low, the persistent issues of vitamin D deficiency, iron deficiency, and childhood obesity require focused attention.

**Conclusion:**

The study highlights ongoing nutritional challenges among Jordanian preschoolers. Although severe anemia was rare, 11% were anemic, and 22.4% had iron deficiency, including 5% with iron deficiency anemia. Vitamin D deficiency affected 22.9%, impacting growth and immunity. While stunting and wasting improved, childhood overweight and obesity rates remained steady. Anemia decreased, but iron deficiency rose by 7%. Despite reduced vitamin A deficiency, stable iron deficiency anemia rates indicate ongoing concerns. Overall, undernutrition is uncommon, but vitamin D and iron deficiencies, along with childhood obesity, need sustained attention and targeted interventions to improve children’s health in Jordan.

## 1 Introduction

Nutrition has significantly improved in the Hashemite Kingdom of Jordan in recent years. Between 1990 and 2012, the prevalence of childhood stunting decreased by roughly two-thirds, and levels of wasting among young children under 5 years old were maintained at a low level, indicating progress in the fight against child undernutrition (1). Since 1996, the ministry of health has implemented a national salt iodization program to combat the burden of micronutrient deficiencies. A survey carried out in 2010 revealed a significant improvement in iodine nutrition among school-age children (2). Additionally, since 2002, wheat flour has been fortified with iron and folic acid. Since then, the program has been expanded to include zinc, vitamins A, and a number of B vitamins (B1, B2, B3, and B12), as well as vitamin D. These changes have significantly decreased the prevalence of severe anemia (3, 4).

Jordan suffers from multiple forms of malnutrition, including undernutrition, micronutrient deficiencies, overweight, obesity and diet-related non-communicable diseases (NCDs). The country has witnessed a rapid nutrition transition due to changing diets and lifestyles, with a shift from undernutrition — but with persistent problems of micronutrient deficiencies — toward greater prevalence of overweight/obesity and diet-related NCDs (5).

These issues now coexist with persistent pockets of undernutrition, low levels of exclusive breastfeeding, alarming levels of low birth weight, and ongoing issues with micronutrient deficiencies (4). Furthermore, Jordan’s food security and malnutrition have been impacted by the COVID-19 pandemic (5). Food security and nutritional health are now in even greater danger due to recent increases in food prices around the world and the conflict in Ukraine (5). Therefore, there is a significant opportunity to improve the health and standard of living for both Jordanians and the numerous refugees who are hosted in the nation.

Jordan has experienced uneven progress in reducing micronutrient deficiencies despite consistent economic growth and declines in some forms of malnutrition. The national micronutrient survey of 2010 found that preschool children and women of childbearing age had high prevalence rates of iron (6) and vitamin D (7), and that about one-fifth of preschoolers had vitamin A deficiency (8). There are conflicting reports regarding the prevalence of anemia.

The prevalence of anemia among preschoolers in Jordan has shown varied reports over time. While the 2010 national survey indicated that about one fifth of preschoolers were affected by anemia, subsequent Population and Family Health Surveys in 2012 and 2017–18 reported that over one third of preschoolers were affected (8, 9). There is inconsistency in anemia prevalence data, with the 2010 national survey reporting one fifth affected, while later surveys in 2012 and 2017–18 found over one third affected (8, 9). Regarding other indicators among preschool age children, stunting exists to a certain extent but is categorized as a ‘low’ public health concern. Wasting and underweight are relatively infrequent occurrences (10). The prevalence of overweight and obesity has been characterized as stable, albeit at slightly elevated levels (11).

Furthermore, our study aims to align with global objectives such as the Sustainable Development Goal (SDG) of achieving zero hunger. By elucidating the severity of micronutrient deficiencies and assessing the double burden of malnutrition, the study findings will contribute directly to the global effort to improve nutrition and food security. By highlighting the socio-economic and geographical frameworks specific to Jordanians, we underscore the significance of our research in addressing local nutritional challenges and informing policy intervention.

Therefore, the Jordan National Micronutrient & Nutrition Survey (JNMNS) 2019 aimed to compare its results with the national micronutrient survey conducted in 2010 and gain a better understanding of the severity of micronutrient deficiencies. The survey also collected simultaneous data on under- and overnutrition to determine the extent of the double burden of malnutrition. Additionally, survey results were contrasted with those of a national survey conducted in 2010 to assess changes in selected key micronutrient intake among preschool children (12). The findings presented in this article are derived from the Jordan National Micronutrient Survey (JNMNS), which was conducted among both Jordanians and Syrian refugees.

## 2 Methods

### 2.1 Sampling approach and sample size determination

The JNMNS 2019 was a national cross-sectional survey with four independent strata. In each of these strata, 40 clusters were selected with equal probability from the list of primary sampling units from the 2015 Jordan census as a result, the entire survey sample will have 160 clusters. In each cluster, a household listing exercise was conducted. In order to achieve sufficient sample size for sub group analyses of Jordanian nationals and other nationalities, different numbers of households were randomly selected in the Northern governorates stratum (20 households) than in the Central, Southern and refugee camps strata (15 households). This resulted in the attempted recruitment of 2,600 households. Given the expected household response rate of 85%, the JNMNS 2019 sample should contain of 2,210 households overall. These 2,210 consenting households have 1,296 eligible preschool children, 1,232 of whom have anthropometry measured and 992 blood specimens collected taking individual response rates into account. The Fisher’s formula for estimating the minimum sample size for prevalence descriptive studies was used as follows:


$$n=\frac{{Z^{2}}_{a/2}P\left(1-P\right)}{d^{2}}*\mathrm{DEFF}*\frac{100}{RR}$$

Where:

n = minimum sample size, expressed as number of units of analysis,
Zα/2 = Z value corresponding to 95% confidence intervals.
P = the assumed prevalence.
d = the allowable sampling error, or ½ the desired confidence interval.
DEFF = design effect.
RR = response rate expressed as a decimal.

### 2.2 Study participants

The study participants were randomly chosen from households within selected primary sampling units. Table 1 outlines the inclusion criteria for survey enrollment, broken down by the target population group. No explicit exclusion criteria were employed, except for the absence of the specified inclusion criteria.

**Table 1:** Inclusion criteria by targeted population group.

Target population	Inclusion criteria
Households	Consent for survey data collection is obtained through oral agreement from the household head, spouse, or another adult member.
Children 0–59 months	Participants must fall within the age range of 0–59 months during the survey (6–59 months specifically for blood sample collection). Individuals need to be recognized as household members by the adults residing in the household. For children within the specified age range, written consent for survey participation must be provided by either the mother or caretaker.

### 2.3 Training of survey teams and field work

The team members were thoroughly trained, and all survey instruments were pre-tested during the training. The training consisted of classroom instruction and practice, and of field testing of all survey procedures. 2 days field pilot testing in non-selected clusters (1 urban cluster, one rural cluster) where each team completed 4–6 households during field testing. In total, 11 teams were deployed to collect data. Each team was composed of one team leader, two interviewers, two phlebotomists, two anthropometrists, and one driver. Additionally, there was a regional supervisor for each stratum who supported coordination of centralization of blood and food specimens at a selected lab.

Two types of questionnaires were distributed to the designated households:

- A household questionnaire is administered to gather information on demographics, socio-economic status, and food purchasing habits within the household.
- An individual questionnaire is utilized for preschool children, with the caregiver serving as the respondent. This questionnaire succinctly captures data on individual health and vaccination status, consumption of micronutrient supplements, knowledge of micronutrients, appropriate feeding practices for infants and young children. Additionally, it records the results from blood collection, encompassing details such as hemoglobin concentration, collection time, and the estimated amount of blood collected through phlebotomy.

### 2.4 Anthropometry and phlebotomy

Anthropometric measurements for children were conducted using established protocols. A scale, capable of subtracting the mother’s weight, was utilized for measuring the children’s weight. In cases where children could not stand quietly on the scale, their weight was taken in the mother’s arms. The height or length of the children was measured using a standardized height board. Additionally, individual data was gathered through interviews and anthropometric measurements. Furthermore, the JNMNS 19 obtained blood specimens from participants who provided their consent during the survey. Experienced phlebotomists collected blood via venipuncture for preschool and school-age children, a 2 mL EDTA-coated tube and a 6 mL trace-element certified tube was used; a complete blood count was done on fresh whole blood, and after centrifugation and aliquoting, serum was shipped to predominantly national and some international laboratories for measurement of the concentrations of various micronutrients and detection of hemoglobinopathies and thalassemia. In preschool children, blood specimens was collected from children aged 6–59 months. Participants identified with severe acute malnutrition or severe anemia were referred for necessary treatment at the nearest health hospital or clinic (13). For more in-depth information, additional details can be referenced in another publication (14).

### 2.5 Measurement and definition of outcomes

The principal nutrition outcomes measured in preschool children are presented in Table 2. Comprehensive information can be found in another publication (14).

**Table 2:** Principal nutrition outcomes measured in preschool children.

Condition measured	Indicator	PSC^a^
Anemia	Hemoglobin concentration^b^	✓
Iron deficiency	Serum ferritin, markers of inflammation (AGP and CRP)	✓
Iron deficiency anemia	Concurrent anemia and iron deficiency measured using ferritin	✓
Vitamin A deficiency	RBP and retinol	✓^c^
Zinc deficiency	Serum zinc	✓
Blood disorders	Sickle cell and α-and β-thalassemia	✓
Vitamin D deficiency	Serum 25[OH]D	✓
Wasting/thinness	Weight-for-height z-score BMI-for-age z-score	✓
Stunting/shortness	Height-for-age z-score Height	✓
Overweight and obesity	Weight-for-height z-score BMI-for-age z-score BMI	✓

^a^PSC, preschool children (0–59 months for anthropometry, 6–59 for blood biomarkers). ^b^A complete blood count was conducted, comprising 22 parameters (e.g., MCV, hematocrit, etc.) ^c^For PSC, serum retinol was measured from all samples.

#### 2.5.1 Anthropometric indicators

In preschool children aged 0–59 months, the assessment of undernutrition (including wasting, stunting, and underweight) and overnutrition followed the criteria outlined in World Health Organization (WHO) Child Growth Standards (15). The z-scores were used to classify children based on specific indicators (16, 17):

- *For wasting*
- Wasting: Z-score less than −2.0 for weight-for-height.
- Moderate wasting: Z-score less than −2.0 but greater than or equal to −3.0.
- Severe wasting: Z-score less than −3.0.
- *For stunting*
- Stunting: Z-score less than −2.0 for height-for-age.
- Moderate stunting: Z-score less than −2.0 but greater than or equal to −3.0.
- Severe stunting: Z-score less than −3.0.
- *For underweight*
- Underweight: Z-score less than −2.0 for weight-for-age.
- Moderate underweight: Z-score less than −2.0 but greater than or equal to −3.0.
- Severe underweight: Z-score less than −3.0.
- *For overweight*
- Overnutrition: Z-score greater than +2.0 for weight-for-height.
- Overweight: Z-score greater than +2.0 but less than or equal to +3.0.
- Obesity: Z-score greater than +3.0

#### 2.5.2 Blood specimens

The cut-off values for biomarkers measured in the JNMNS 2019 are presented in Table 3.

**Table 3:** Clinical cut-off points and classifications for biomarker indicators.

	Adequate	Mild	Moderate	Severe
Hemoglobin (18) PSC: Children 6–59 months	≥ 110 g/L	100–109 g/L	70–99 g/L	<70 g/L
	Deficiency cut-offs			
RBP and retinol (19, 20)	≤0.7 μM/L*			
Serum ferritin (21)	< 12 μg/L*			
α1-acid-glycoprotein (22)	>1 g/L			
C-reactive protein (23)	>5 mg/L			
25[OH]D (24)	<12 ng/mL, deficiency; <20 ng/mL, insufficiency			
Serum zinc (25) Children 6–59 months	Morning, non-fasting: 65 μg/dL, afternoon, non-fasting: 57 μg/dL			

*Adjusted for sub-clinical inflammation using suitable algorithms (26, 27), it is important to highlight that there are no established thresholds for Retinol-Binding Protein (RBP). Nevertheless, given the strong correlation between serum retinol and RBP, the same threshold was applied.

The cut-off defining normal hemoglobin concentrations was adjusted for altitude of residence (Table 4).

**Table 4:** Adjustments in cut-off defining anemia, by altitude of residence (18).

Altitude (meters)	Increase in cut-off point defining anemia (g/L)
< 1,000	No adjustment
1,000–1,249	+ 2
1,250–1749	+ 5
1750–2,249	+ 8

#### 2.5.3 Blood specimens

The methods of hemoglobin, serum ferritin, C-reactive protein and alpha-1 acid glycoprotein, Retinol, Retinol binding Protein (RBP), Vitamin D and Zinc analysis have explained in details elsewhere (14).

#### 2.5.4 Data management and analysis

Data collection was executed electronically, employing tablet computers equipped with the Open Data Kit (ODK) software. Continuous monitoring of the data was undertaken to ensure frequent quality control. Descriptive statistics for key indicators were computed, stratified by different characteristics and strata.

#### 2.5.5 Ethical considerations

To ensure adherence to principles safeguarding survey participants and minimizing potential risks, ethical approval for this study was secured from the Institutional Review Board at Jordan University of Science & Technology. Selected caregivers of children were required to furnish written informed consent for themselves and their wards. In cases where participants could not read or write, the consent form was read aloud, and a thumbprint or fingerprint was obtained as proof of agreement, serving as a substitute for a signature. Alternatively, participants could designate an alternative to sign on their behalf. It was clearly communicated to respondents that they retained the freedom to withdraw from the survey at any stage, even after providing written consent.

Rigorous confidentiality measures were precisely maintained throughout the entire process of data collection, processing, and analysis. For infants below 6 months, no blood samples were collected to avoid causing unnecessary discomfort. Participants diagnosed with severe acute malnutrition were promptly referred to a nearby health facility.

### 2.6 Data analysis

Data analysis was done using SPSS version 26. Data analysis included calculation of proportions to derive the prevalence of dichotomous outcomes and calculation of mean and median averages for continuous outcomes. Nationwide prevalence estimates were calculated by using weighted analysis to account for the unequal probability of selection in the 3 strata. The statistical precision of all estimates was assessed using 95% confidence limits. All measures of precision, including confidence limits and chi square *p* values for differences, were calculated accounting for the complex cluster and stratified sampling used by the JNMNS 2019.

## 3 Results

### 3.1 Characteristics

Table 5 provides an overview of the demographic attributes of preschool children engaged in the JNMNS. The majority of these children are situated in the Northern stratum. Approximately half of the children are male, and an overwhelming 85% of them inhabit urban households.

**Table 5:** Description of sampled preschool age children (0–59 months), settled population, Jordan.

Characteristic	*n*	%^a^	(95% CI)^b^
Age Group (in months)			
0–5	91	11.7	(9.1, 14.8)
6–11	77	9.7	(7.3, 12.8)
12–23	156	20.4	(17.1, 24.1)
24–35	172	22.0	(18.5, 26.0)
36–47	150	17.1	(14.0, 20.8)
48–59	164	19.1	(16.3, 22.2)
Sex			
Male	399	50.1	(44.7, 55.4)
Female	411	49.9	(44.6, 55.3)
Residence			
Urban	647	85.0	(73.2, 92.2)
Rural	161	15.0	(7.8, 26.8)
Stratum			
Central	187	23.1	(16.0, 32.2)
Northern	355	43.8	(33.5, 54.7)
Southern	268	33.1	(23.9, 43.7)

The n’s are un-weighted numbers in each subgroup; the sum of subgroups may not equal the total because of missing data. ^a^Percentages weighted for unequal probability of selection. ^b^CI, confidence interval calculated taking into account the complex sampling design.

### 3.2 Anemia, iron deficiency, and iron deficiency anemia

**Figure 1:**
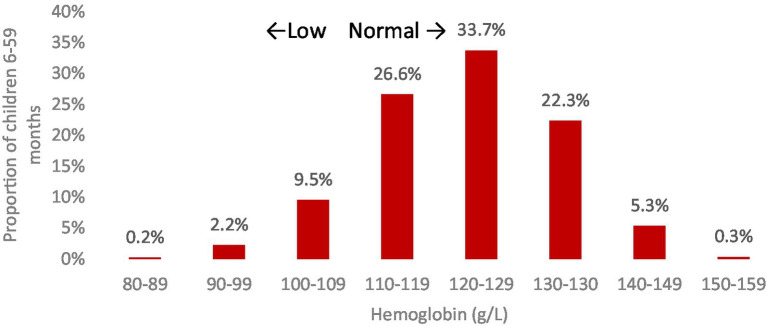
The distribution of hemoglobin concentrations (g/L) in children aged 6–59 months, residing in settled areas of Jordan. *Significantly significant difference (*p* < 0.05).

Nationally, approximately 12% of preschool children were found to be anemic, with all cases classified as moderate or mild; no instances of severe anemia were identified (refer to Table 6). According to the WHO standards (18), this level of anemia prevalence is considered a mild public health concern. Significant associations with anemia were observed for age group and wealth quintile, with a notably higher prevalence among male children and those aged 6–11 months. The distribution of hemoglobin values for children, illustrated in Figure 1, is roughly symmetric, with the majority of values above the 110 g/L cutoff point. The mean hemoglobin concentration among settled children aged 6–59 months was 122.2 g/L (95%CI 121.8, 123.6 g/L).

**Table 6:** Severity of anemia in children 6–59 months, by various demographic characteristics, settled population, Jordan.

Characteristic	Mild anemia^b^			Moderate anemia^b^			Severe anemia^b^		
	*n*	%^a^	95% CI^c^	*n*	%^a^	95% CI^c^	*n*	%^a^	95% CI^c^
Total	44	9.5	(6.4, 13.8)	13	2.4	(1.1, 5.2)	0	0.0	–
Age (in months)									
6–11	6	26.8	(11.3, 51.3)	3.0	11.7	(2.8, 38.1)	–	–	–
12–23	12	9.8	(4.7, 19.5)	5.0	5.1	(1.4, 17.2)	–	–	–
24–35	12	9.3	(4.4, 18.5)	1.0	0.6	(0.1, 4.7)	–	–	–
36–47	10	9.1	(3.8, 20.0)	1.0	0.4	(0.0, 2.6)	–	–	–
48–59	4	4.8	(1.3, 15.9)	3.0	1.5	(0.4, 5.3)	–	–	–
Sex									
Male	22	12.2	(7.3, 19.6)	8.0	3.7	(1.3, 9.8)	–	–	–
Female	22	6.9	(4.0, 11.5)	5.0	1.2	(0.5, 3.1)	–	–	–
Residence									
Urban	38	9.4	(6.1, 14.2)	12	2.7	(1.2, 5.9)	–	–	-
Rural	6.0	10.1	(5.6, 17.4)	1	0.6	(0.1, 4.6)	–	–	–
Stratum									
Central	7	7.8	(3.8, 15.3)	2	2.2	(0.6, 8.3)	–	–	–
Northern	20	11.9	(7.6, 18.2)	3	1.8	(0.6, 5.4)	–	–	–
Southern	17	11.5	(6.8, 18.8)	8	5.4	(2.7, 10.5)	–	–	–
Wealth quintile									
Poorest	20	12.3	(6.9, 21.0)	10	6.3	(2.7, 14.1)	–	–	–
Second	9	9.6	(4.7, 18.6)	3	3.3	(0.7, 15.4)	–	–	–
Middle	5	5.1	(2.2, 11.5)		0.0	–	–	–	–
Fourth	6	5.1	(1.4, 16.5)		0.0	–	–	–	–
Wealthiest	4	19.6	(7.7, 41.6)		0.0	–	–	–	–

The “*n*’s” represent the numerators for a particular sub-group. ^a^All percentages, excluding region-specific estimates, are weighted to account for unequal probability of selection among strata. ^b^Mild, moderate, and severe anemia are defined as hemoglobin levels of 100–109 g/L, 70–99 g/L, and < 70 g/L, respectively. ^c^CI, confidence interval, calculated considering the complex sampling design.

Iron deficiency was noted in approximately 26% of preschool children, and significant associations with age group and wealth quintile were observed for this condition as well. Five percent of preschool children exhibited concurrent anemia and iron deficiency, commonly known as iron deficiency anemia (IDA). Variations were noted by age group, particularly with children aged 6–11 months showing significantly higher proportions of IDA (17.0%) compared to other age groups (2–7%).

**Figure 2:**
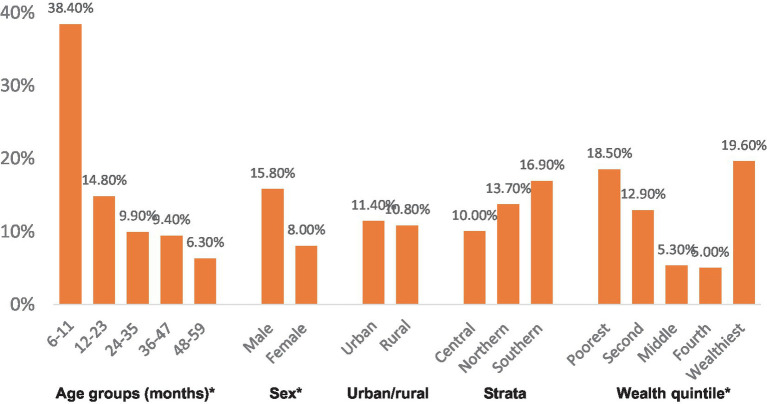
Prevalence of anemia Strata. *Significantly significant difference (*p* < 0.05).

**Figure 3:**
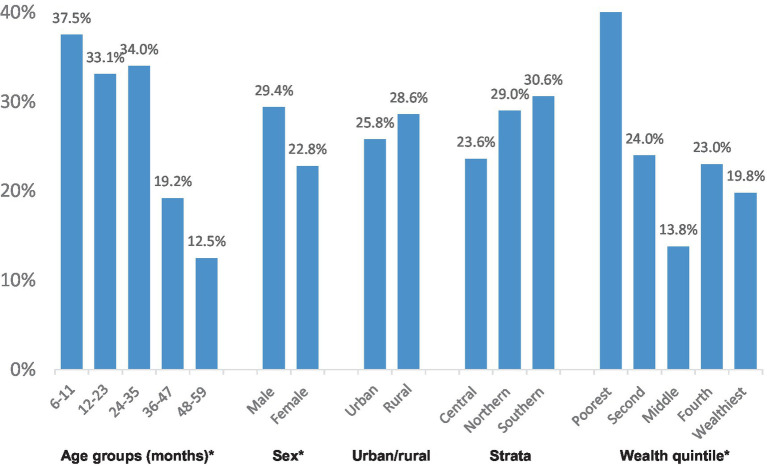
Prevalence of iron deficiency. *Significantly significant difference (*p* < 0.05).

**Figure 4:**
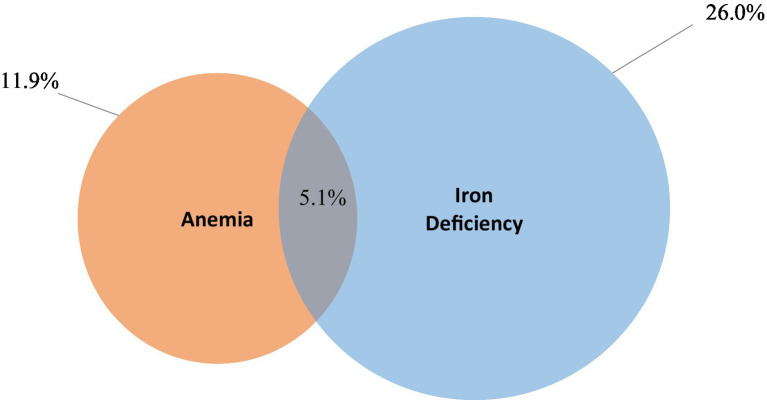
Prevalence of iron deficiency anemia.

Figures 2–4 show the prevalence of anemia, iron deficiency, and iron deficiency anemia in children aged 6–59 months, categorized by various demographic characteristics within the settled population.

### 3.3 Vitamin A deficiency

**Figure 5:**
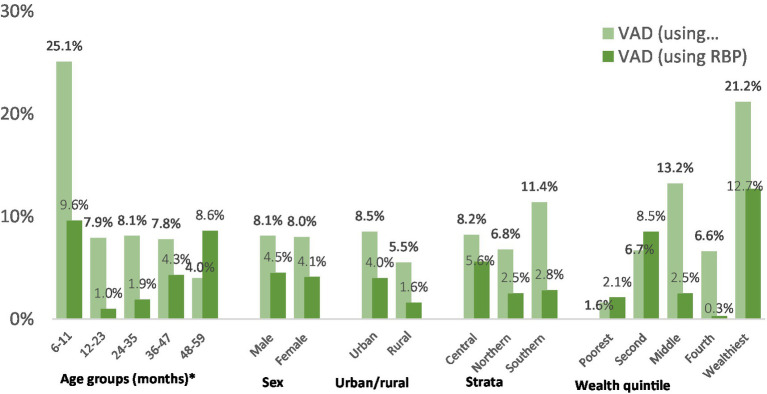
The prevalence of vitamin A deficiency in children aged 6–59 months within the settled population of Jordan is detailed across various demographic characteristics. *Significantly significant difference (*p* < 0.05).

Approximately 4–8% of preschool children were identified as having vitamin A deficiency, with the prevalence depending on the indicator used—either serum retinol or RBP. Regardless of the chosen indicator, the observed prevalence of vitamin A deficiency is considered a mild public health concern on a national level, as per the World Health Organization (WHO) criteria (28). Significant variations were noted based on age and wealth quintile (Figure 5).

**Figure 6:**
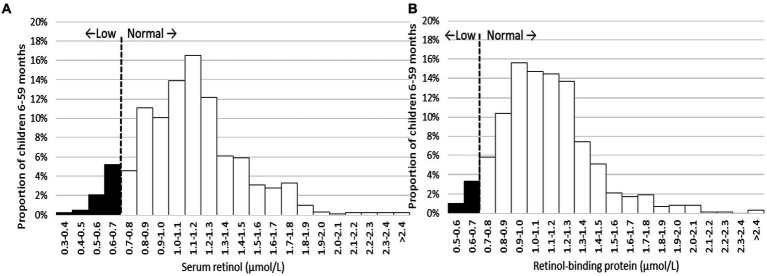
Distribution of serum retinol **(A)** and inflammation-adjusted retinol-binding protein **(B)** in children 6–59 months, settled population, Jordan.

Figure 6 illustrates the distribution of retinol binding protein (RBP) and serum retinol values for children in the settled population. Notably, the majority of values for both indicators surpass the threshold of 0.7 μmol/L. It’s important to note that the histogram for RBP is not inflammation-adjusted, resulting in more cases falling below the threshold. The mean serum retinol concentration in this group was 1.13 μmol/L (95%CI: 1.08, 1.16), and the mean inflammation-adjusted retinol binding protein concentration was 1.13 μmol/L (95%CI: 1.09, 1.16).

### 3.4 Vitamin D deficiency and insufficiency

**Figure 7:**
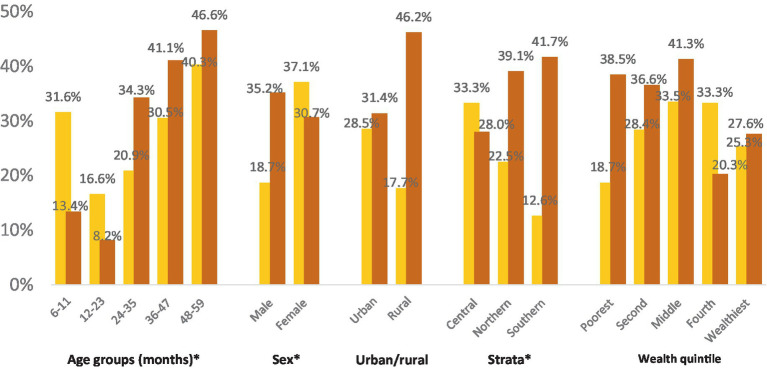
Vitamin D deficiency and insufficiency prevalence in children 6–59 months within the settled population of Jordan across different demographic characteristics. *Significantly significant difference (*p* < 0.05).

Approximately one-third of children aged 6–59 months within the settled population were found to be vitamin D deficient, while another third exhibited vitamin D insufficiency, resulting in nearly two-thirds of children having inadequate vitamin D status (refer to Figure 7). Apart from the 6–11 months’ age group, which displayed a high prevalence of inadequate vitamin D status, a discernible pattern emerged, indicating an increasing prevalence with age. Additionally, a gender disparity was observed, with a higher proportion of girls experiencing vitamin D deficiency compared to boys. Notably, children in the Southern stratum exhibited a lower frequency of vitamin D deficiency compared to those in the Central or Northern strata. Other demographic factors did not exhibit a clear association with vitamin D status.

**Figure 8:**
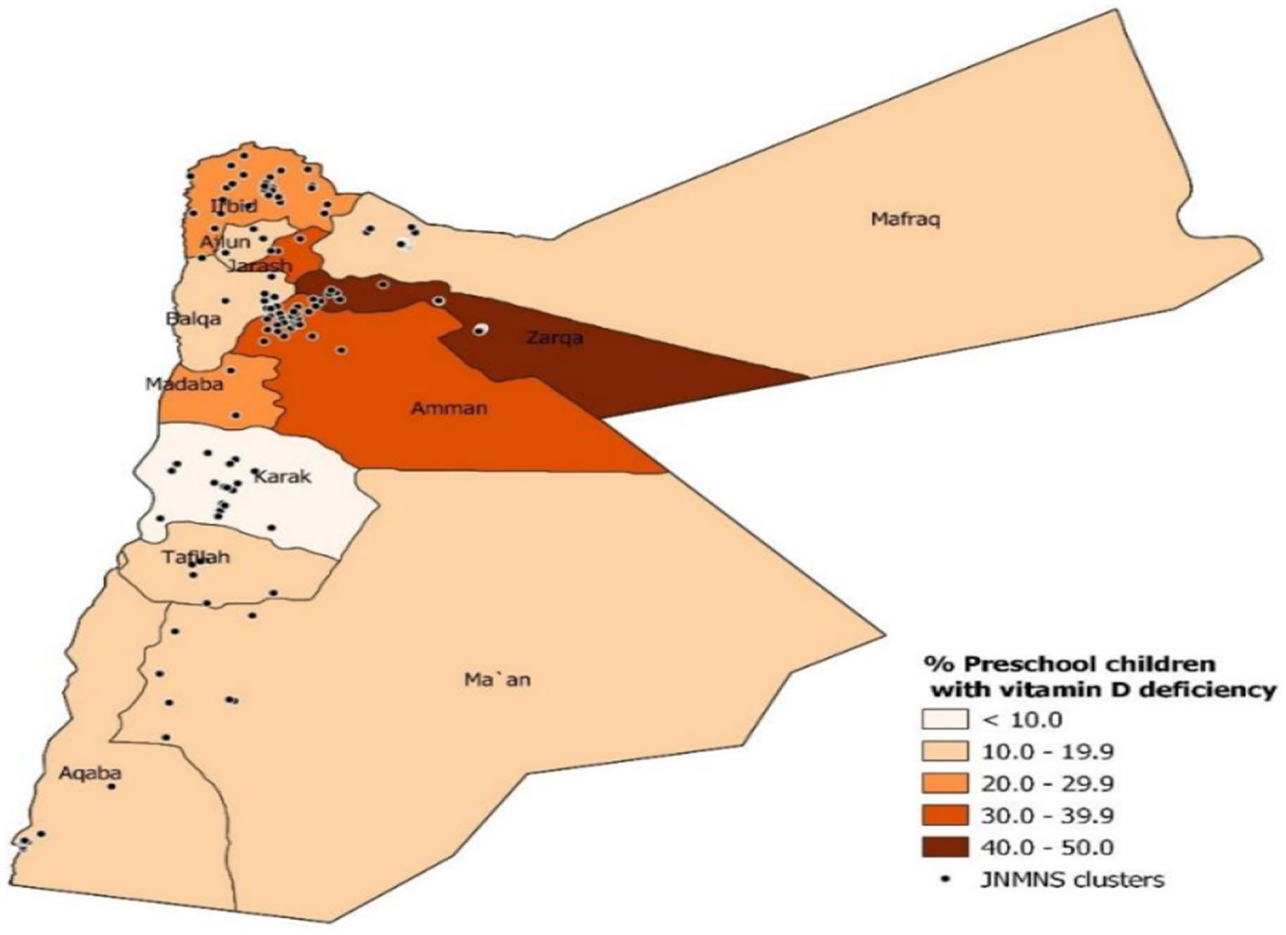
The prevalence of vitamin D deficiency (defined as <12 ng/mL) in preschool children within the settled population of Jordan defined by governorate.

The geometric mean of vitamin D concentration was 16.8 ng/mL (95%CI: 15.7, 17.9). Figure 8 illustrates the geographic distribution of vitamin D deficiency among preschool children in the settled population, revealing higher prevalence rates in the Northern and Central strata compared to the South. Karak stands out as an exception, with a prevalence below 10%.

### 3.5 Zinc deficiency

**Figure 9:**
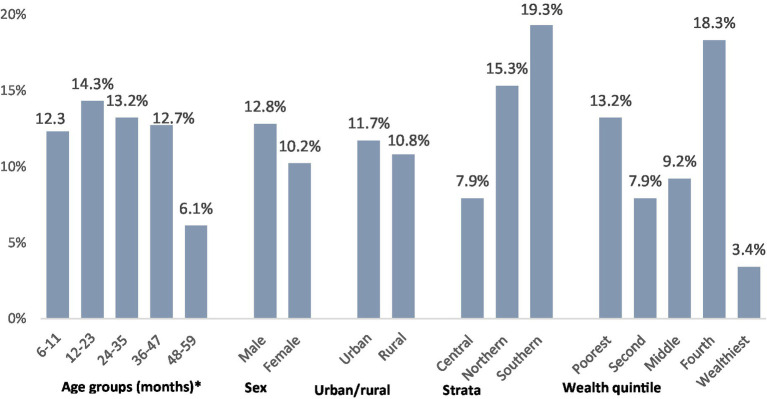
Zinc deficiency prevalence in children aged 6–59 months, categorized by various demographic characteristics within the settled population of Jordan. *Significantly significant difference (*p* < 0.05).

Zinc deficiency affected slightly more than 1 out of 10 preschool children in the settled population. Significant variations were observed by age, with older children exhibiting a significantly lower prevalence. However, no statistically significant differences in zinc deficiency were found between male and female children, or among children living in different residences, strata, or households with different wealth levels (refer to Figure 9). The mean serum zinc level in this group was 72.2 μg/dL (95%CI: 69.7, 74.7).

### 3.6 Stunting

**Figure 10:**
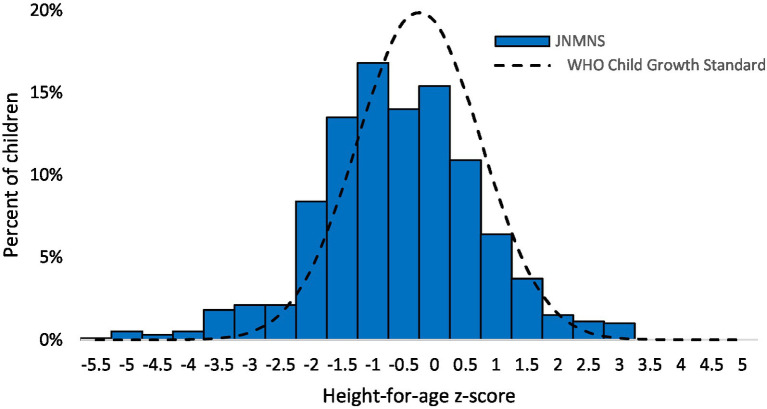
Height-for-age z-scores distribution in children <5 years of age, settled population, Jordan.

Regarding stunting, the overall prevalence is 7.4% among preschool children in the settled population, as indicated in Table 7. This prevalence is relatively low, considering recently updated thresholds that define categories for stunting into public health relevance (29). Significant variations were noted by stratum, with fewer children affected in the Southern stratum. No other demographic subgroup analysis identified statistically significant differences. While not reaching statistical significance, children of mothers with short stature were twice as likely to be stunted and four times as likely to be severely stunted compared to children whose mothers had normal stature. The distribution of height-for-age z-scores, illustrated in Figure 10, is slightly shifted to the left. The mean height-for-age z-score was −0.33 (95% CI: −0.42, −0.24), with a standard deviation (SD) of 1.26.

**Table 7:** Prevalence of stunting in children 0–59 months, categorized by various demographic characteristics within the settled population of Jordan.

		Severely stunted^b^		Moderately stunted^b^			Total stunted^c^		
Characteristic	*n*	%^a^	95% CI	%	95% CI	*p*-value ^d^	%	95% CI	*p*-value^d^
Total	750	3.2	(1.8, 5.8)	4.2	(2.7, 6.5)		7.4	(5.1, 10.7)	
Age (in months)									
0–11	155	6.6	(2.7, 15.0)	3.1	(1.1, 8.6)	0.491	9.7	(5.1, 17.7)	0.581
12–23	147	1.7	(0.6, 4.6)	2.8	(0.8, 9.1)		4.4	(1.9, 10.0)	
24–35	157	3.1	(0.8, 11.5)	6.0	(2.7, 12.6)		9.1	(4.8, 16.8)	
36–47	138	2.1	(0.4, 11.2)	6.1	(2.6, 14.0)		8.2	(3.0, 20.5)	
48–59	53	2.3	(0.5, 10.7)	3.2	(1.0, 9.6)		5.5	(2.2, 13.2)	
Sex									
Male	362	3.0	(1.4, 6.4)	4.0	(2.1, 7.5)	0.911	7.0	(4.5, 10.8)	0.699
Female	388	3.5	(1.6, 7.5)	4.4	(2.6, 7.3)		7.9	(4.7, 13.1)	
Residence									
Urban	601	3.7	(2.0, 6.7)	4.1	(2.5, 6.8)	0.281	7.8	(5.2, 11.6)	0.493
Rural	147	0.9	(0.2, 4.5)	5.0	(2.1, 11.2)		5.9	(2.8, 11.9)	
Stratum									
Central	172	4.7	(2.3, 9.2)	4.1	(2.0, 8.0)	0.042	8.7	(5.2, 14.2)	0.142
Northern	337	1.5	(0.6, 3.4)	4.7	(2.8, 8.0)		6.2	(3.9, 9.8)	
Southern	241	0.8	(0.2, 3.2)	2.9	(1.5, 5.5)		3.7	(1.8, 7.4)	
Wealth quintile									
Poorest	213	5.7	(2.6, 11.9)	6.6	(4.0, 10.9)	0.081	12.3	(7.9, 18.7)	0.103
Second	168	1.0	(0.2, 4.0)	2.2	(0.6, 7.8)		3.2	(1.2, 8.1)	
Middle	165	0.6	(0.1, 2.8)	5.9	(2.5, 13.7)		6.5	(2.9, 14.1)	
Fourth	126	6.4	(1.8, 20.2)	3.3	(0.9, 11.0)		9.7	(4.0, 21.7)	
Wealthiest	78	2.5	(0.4, 16.1)	0.8	(0.1, 5.5)		3.3	(0.7, 14.7)	
Mother’s stature									
Short (< 150 cm)	24	16.6	(4.6, 44.9)	0.0	--	0.112	16.6	(4.6, 44.9)	0.310
Normal (> 150 cm)	321	4.5	(1.9, 10.2)	3.8	(1.8, 7.8)		8.3	(4.7, 14.3)	

The “*n*’s” represent unweighted numbers (denominator) for each subgroup; subgroups that do not sum to the total indicate missing data. ^a^Percentages are weighted to account for unequal probability of selection. ^b^Severe stunting is defined as having a height-for-age z-score below −3 standard deviations from the WHO Child Growth Standards population median; moderate stunting is defined as having a height-for-age z-score equal to or above −3 standard deviations and less than −2 SD from the WHO Child Growth Standards population median. ^c^Total stunting includes both severely and moderately stunted children. ^d^A p-value <0.05 indicates that at least one subgroup is significantly different from the others. Chi-square results are based on total stunting.

### 3.7 Wasting and underweight

**Figure 11:**
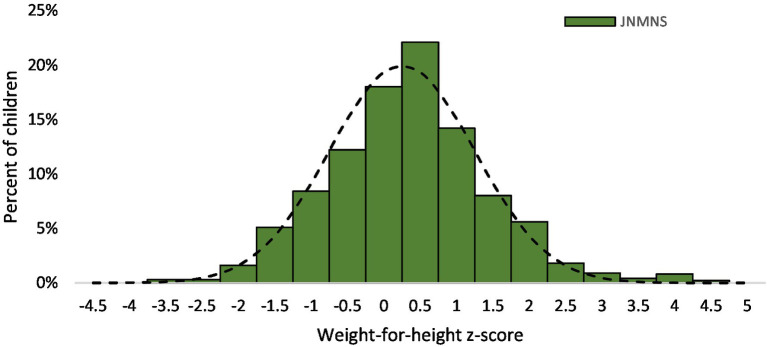
Weight-for-height z-scores distribution in children 6–59 months, settled population, Jordan.

The prevalence of wasting among preschool children in the settled population was only 0.6% (95% CI: 0.2, 2.0). The small number of affected children limits the feasibility of conducting meaningful subgroup analyses (Figure 11).

Similarly, the prevalence of underweight in this population group was very low, affecting 2.7% (95% CI: 1.6, 4.7) of children. Only 0.6% of children were identified with severe underweight. Due to the limited number of affected children, no subgroup analyses were performed.

### 3.8 Overweight and obesity

The Prevalence of overweight among children under 5 years was 9.2% in 2019 (2.2% with obesity). Prevalence was higher among boys (11.7%) than girls (6.7%), in urban and rural areas (10% vs. 5.2%) and in wealthier households (14.2% vs. 4.8% in the wealthiest compared to the poorest income quintile) as shown in Table 8.

**Table 8:** Prevalence of overweight and obesity in children 0–59 months, by various demographic characteristics, settled population, Jordan.

		Obesity^b^		Overweight^b^			Total overweight or obesity^b^		
Characteristic	*n*	%^a^	95% CI	%	95% CI	*p*-value^c^	%	95% CI	*p*-value^c^
Total	747	2.2%	(1.2, 4.0)	7.0%	(4.9, 9.7)		9.2%	(6.9, 12.1)	
Age (in months)									
0–11	153	2.0%	(0.4, 9.4)	10.6%	(5.9, 18.1)	0.630	12.6%	(7.5, 20.4)	0.328
12–23	147	3.3%	(1.1, 9.0)	7.1%	(3.6, 13.4)		10.4%	(6.0, 17.4)	
24–35	156	2.9%	(0.9, 9.2)	7.6%	(3.8, 14.8)		10.5%	(5.7, 18.6)	
36–47	138	1.7%	(0.6, 5.2)	5.0%	(1.7, 14.0)		6.7%	(2.9, 15.1)	
48–59	153	1.1%	(0.3, 4.3)	3.8%	(1.3, 10.6)		4.8%	(2.0, 11.4)	
Sex									
Male	363	2.3%	(1.1, 4.8)	9.4%	(6.5, 13.5)	0.072	11.7%	(8.6, 15.7)	0.023
Female	384	2.1%	(0.8, 5.4)	4.5%	(2.6, 7.7)		6.7%	(4.2, 10.5)	
Residence									
Urban	596	2.5%	(1.4, 4.6)	7.5%	(5.2, 10.6)	0.286	10.0%	(7.5, 13.2)	0.204
Rural	149	0.7%	(0.1, 4.6)	4.5%	(1.5, 12.5)		5.2%	(1.8, 13.7)	
Stratum									
Central	171	1.8%	(0.6, 5.2)	5.8%	(3.2, 10.6)	0.397	7.6%	(4.6, 12.3)	0.266
Northern	333	3.3%	(1.8, 5.9)	8.7%	(6.0, 12.5)		12.0%	(8.7, 16.3)	
Southern	243	1.2%	(0.4, 3.9)	7.4%	(4.2, 12.6)		8.6%	(5.1, 14.1)	
Wealth quintile									
Poorest	213	1.3%	(0.4, 4.5)	3.5%	(1.4, 8.3)	0.477	4.8%	(2.3, 9.9)	0.310
Second	165	3.1%	(1.0, 9.5)	6.2%	(2.9, 12.8)		9.3%	(4.8, 17.0)	
Middle	164	0.9%	(0.2, 3.6)	9.3%	(5.2, 16.2)		10.2%	(5.8, 17.2)	
Fourth	128	3.3%	(0.9, 11.0)	7.4%	(3.3, 15.5)		10.7%	(5.6, 19.4)	
Wealthiest	77	3.7%	(0.8, 15.3)	10.5%	(4.4, 22.8)		14.2%	(6.7, 27.5)	

The *n*’s are un-weighted numbers (denominator) for each subgroup; subgroups that do not sum to the total have missing data. ^a^Percentages weighted for unequal probability of selection. ^b^Overweight is defined as having a weight-for-height z-score greater than +2 but less than or equal to +3 standard deviations from the WHO Child Growth Standards population median; obesity is defined as having a weight-for-height z-score greater than +3 standard deviations from the WHO Child Growth Standards population median. ^c^*P* value <0.05 indicates that at least one subgroup is significantly different from the others.

### 3.9 Infant and young child feeding indicators and supplements

**Figure 12:**
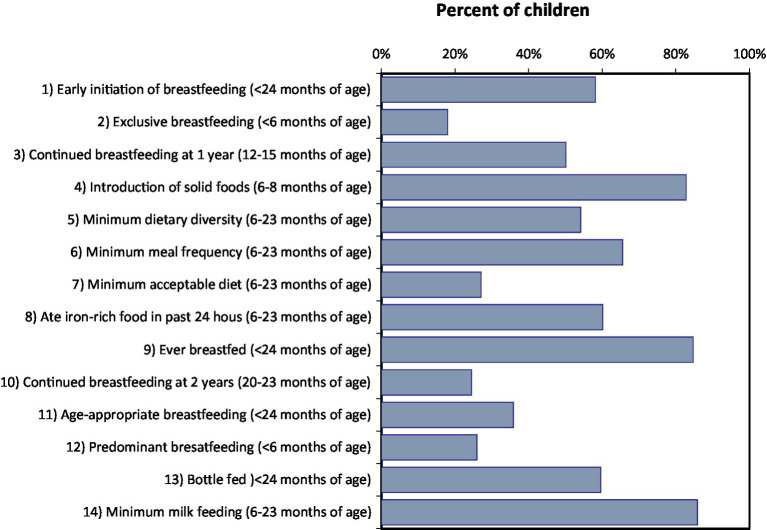
Prevalence of standard WHO/UNICEF infant and young child feeding indicators in children of various ages, settled population, Jordan.

According to WHO/UNICEF recommendations (16), Figure 12 summarizes the various infant and young child feeding indicators. Ever breastfeeding was widespread, but less than two thirds of parents initiated breastfeeding at an early age. In children under the age of 6 months, exclusive breastfeeding is very low below the WHO recommendations. Furthermore, continuing breastfeeding after 1 year and after 2 years is not very common. A relatively brief breastfeeding period is indicated by the median breastfeeding duration of 14 months among children under 24 months (data not shown).

The indicators of complementary feeding, such as the introduction of solid foods, the minimum requirements for dietary diversity, meal frequency, and dietary acceptability are reasonable but not ideal. As presented in Table 9, nearly two thirds of kids had consumed iron-rich foods or taken iron supplements within the previous day before data collection. Approximately two thirds of kids had food from a bottle in the previous 24 h, and breastfeeding that is age-appropriate and prevalent is relatively poor. Water, infant formula, and other non-human milks were the most frequently consumed liquids by infants younger than 6 months. In the previous 24 h, nearly half of kids aged 6 to 23 months had consumed sugary foods or drinks. A third of the 6-to 23-month-old children consumed fried or salty snacks.

**Table 9:** Additional dietary indicators in children less than 24 months of age, settled population, Jordan.

Characteristic	*N*	%^a^	95% CI^b^
Liquids other than breast milk consumed in past 24 h (<6 months of age)			
Plain water	42	49.7%	(35.5, 63.9)
Infant formula	37	42.0%	(27.2, 58.3)
Tinned, powdered, or other non-human milk	35	41.4%	(28.9, 55.1)
Juice or juice drinks	6	3.8%	(1.7, 8.3)
Shourba or clear broth	7	4.1%	(1.9, 8.6)
Yogurt	10	7.8%	(3.3, 17.3)
Thin porridge	4	4.0%	(1.0, 15.4)
Other liquids	9	4.6%	(2.3, 9.3)
Ate sugary foods in past 24 h (6–23 months of age)			
Yes	118	46.9%	(37.2, 56.9)
No	115	53.1%	(43.1, 62.8)
Consumed sugary drinks in past 24 h (6–23 months of age)^c^			
Yes	103	38.8%	(31.3, 46.9)
No	130	61.2%	(53.1, 68.7)
Ate salty/fried foods in past 24 h (6–23 months of age)^d^			
Yes	90	37.5%	(29.7, 46.0)
No	143	62.5%	(54.0, 70.3)

The *n*’s are un-weighted numbers for each subgroup; subgroups that do not sum to the total have missing data. ^a^Percentages weighted for unequal probability of selection. ^b^CI = confidence interval calculated taking into account the complex sampling design. ^c^The question was about ‘Sugar-sweetened beverages (soft drinks/fizzy drinks, chocolate drinks, malt drinks, yoghurt drinks, sweet tea or coffee with sugar, sweetened fruit juices and “juice drinks”). ^d^The question was about ‘Salty or fried snacks: Crisps and chips, fried dough or other fried snacks (e.g., Samposic).

**Figure 13:**
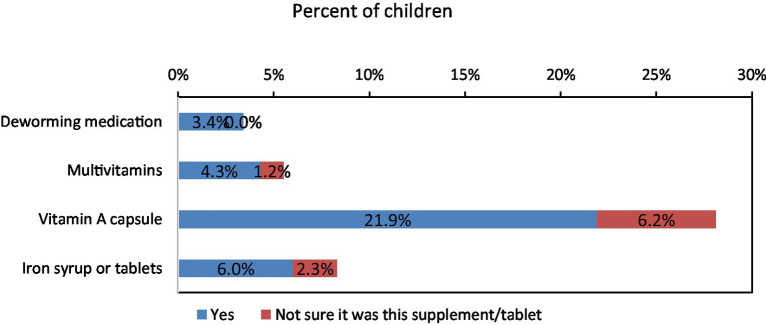
Percentages of children under 5 years of age who have taken different supplements in past 6 months, settled population, Jordan.

Although almost one quarter of children consumed vitamin A supplements, supplement consumption in general and treatment with an anthelmintic drug in the preceding 6 months was low in this age group (Figure 13).

## 4 Discussion

The JNMNS 2019 aimed to compare its findings with the 2010 national survey to understand micronutrient deficiencies better. It also assessed the double burden of malnutrition by collecting data on both under-and overnutrition. Additionally, the survey evaluated changes in key micronutrient intake among preschool children compared to the 2010 survey.

Data from 2019 suggest that anemia among young children is a mild public health problem in the settled population. Among young children aged between 6 and 59 months, 11.9% were anemic. No children in this age group had severe anemia and 2.4% had moderate anemia. In the same age group, 26% were iron deficient and 5.1% had iron-deficiency anemia in 2019. Not all anemia in Jordan is due to iron deficiency. As children get older, there is less overlap between anemia and iron deficiency, suggesting that other causes of anemia are more significant. Overall, anemia prevalence in this age group decreased between 2010 and 2019, whereas iron deficiency prevalence increased and anemia caused by iron deficiency remained largely unchanged.

The observed declines in overall anemia and severe anemia may be attributed to some extent to the successful national wheat flour fortification program. Given the substantial difference in prevalence of anemia and iron-deficiency anemia in some groups, further research is needed to investigate the potential causes of anemia and iron deficiency. An analysis of these data will be able to inform policy responses and intervention. The most recent JNMNS 2019 looked into factors related to anemia and iron deficiency.

The 2010 Jordan Micronutrient Survey recorded a lower prevalence of iron deficiency. However, due to the incomplete correction for inflammation in serum ferritin concentrations, the computed values likely leaned toward higher levels, resulting in an underestimated prevalence of iron deficiency. A meta-analysis for the Middle East and North Africa region indicated that 35% of anemia can be attributed to iron deficiency (30). Our preliminary analysis of anemia risk factors suggests that both iron and vitamin A deficiency play significant roles, while the scarcity of α-and β-thalassemia minimizes their impact on public health.

The prevalence of vitamin A deficiency among young children under 5 is categorized as a mild public health issue in Jordan. According to 2019 data, the prevalence was 4.8% among preschool children, with variations depending on the indicator used (8.1% based on serum retinol and 4.3% based on RBP). This represents an improvement since 2010, possibly attributed to the intensified vitamin A supplementation program implemented since 2012. However, it’s important to note that the 2010 survey excluded children aged 6 to 11 months, a group identified by the JNMNS as having a considerably higher prevalence of vitamin A deficiency compared to older children.

In Jordan, a substantial proportion of young children are vitamin D deficient. Studies in the Middle East and North Africa region reported varying prevalence rates for vitamin D deficiency in this age group, ranging from 12 to 60% (31).

The prevalence of zinc deficiency in preschool children within the settled population of Jordan is lower than the global levels reported by the WHO Vitamin and Mineral Nutrition Information System (VMNIS) (32, 33). The JNMNS found slight association between stunting and zinc deficiency, possibly attributable to the low prevalence of both zinc deficiency and stunting.

Overall, the prevalence of undernutrition in young Jordanian children is low, with a reduction in stunting between 2010 and 2019. Stunting prevalence in the settled population is considered low according to WHO cut-off values, constituting a moderate public health problem. The prevalence of overweight among children under 5 years was 9.2% in 2019 (2.2% with obesity), signifying a moderate public health issue that demands policy attention (34).

To mitigate overweight and obesity, WHO recommends limiting energy intake from fats and sugars and engaging in regular physical activity (35). In the JNMNS, almost half of children aged 6–23 months consumed sugary foods, one-third consumed sugary drinks, and over one-third consumed salty/fried snacks in the previous 24 h. Additionally, very few children participated in organized physical activity, though no association was found between physical activity and overweight/obesity in young children.

## 5 Strengths and limitations of the JNMNS

One of the study limitation is the response rate among preschool children which was notably lower than expected. This was primarily due to a relatively high rate of refusal among this age group and the challenges associated with performing phlebotomy on children under 2 years old. Although the study provided national estimates with confidence intervals deemed satisfactory for preschool children, the relatively small sample size of young children resulted in lower precision than expected for subgroup-specific estimates based on laboratory test results.

The trends in various nutrition indicators did not follow a consistent pattern. The prevalence of anemia decreased between 2010 and 2019 in both preschool children and non-pregnant women. However, during the same period, the prevalence of iron deficiency increased, and the prevalence of iron deficiency anemia remained stable. Additionally, there was a decline in the prevalence of vitamin A deficiency, while vitamin D deficiency remained persistently high without significant changes.

## 6 Conclusion and recommendations

Overall, stunting was less common in this age group between 2010 and 2019, with a low prevalence of under-nutrition among young Jordanian children in the settled population, which falls within the WHO’s cut-off values for public health significance.

The following WHO recommendations should be enforced for feeding infants and young children (6–23 months): Continued breastfeeding, introduction of solid, semisolid, or soft foods at 6 months, ensuring food diversity (at least five food groups per day), maintaining appropriate meal frequency (two to three times per day between 6 and 8 months, increasing to three to four times per day between 9 and 23 months, with nourishing snacks offered once or twice a day as desired), safe food preparation, and responsive feeding based on infants’ cues are all crucial.

For children aged 2–5 years, promoting a healthy and balanced diet in households, kindergartens, and schools, along with the activation of growth monitoring at Primary Health Centers (PHCs) and schools, is essential.

Anemia in Jordan has a complex etiology involving both nutritional and non-nutritional factors, making it a multifactorial condition. Addressing anemia should consider additional potential contributing factors beyond iron deficiency. While Jordan may not be on track to achieve the WHA and SDG goals, anemia is shifting from severe to moderate to mild among preschool children, which reflects the impact of the national flour fortification program.

The prevalence of vitamin A deficiency among young children under 5 is classified as a mild public health problem in Jordan. This reflects the diversity in the diets of this group, as well as the impact of wheat flour fortification. However, vitamin D deficiency remains a concern, and there is a need to encourage sun exposure and consider increasing the levels of vitamin D3 in flour fortification to align with WHO recommendations.

The implementation of the national nutrition strategy by the Ministry of Health (MOH) using a multisectoral approach in collaboration with significant stakeholders is expected to yield more effective results and outcomes. Additionally, it is essential to integrate comprehensive nutrition and health education for parents into the national nutrition strategy to effectively enhance children’s nutritional status. This education can empower parents with the knowledge and tools needed to make informed decisions about their children’s diet and health.

## Data availability statement

The datasets presented in this study can be found in online repositories. The names of the repository/repositories and accession number(s) can be found at: https://www.moh.gov.jo/ebv4.0/root_storage/ar/eb_list_page/jnmns19_report_220207_printable.pdf.

## Ethics statement

The studies involving humans were approved by Ministry of Health, Jordan, Amman. The studies were conducted in accordance with the local legislation and institutional requirements. Written informed consent for participation in this study was provided by the participants’ legal guardians/next of kin.

## Author contributions

RB: Conceptualization, Data curation, Formal analysis, Funding acquisition, Methodology, Project administration, Supervision, Validation, Writing – original draft, Writing – review & editing. RT: Writing – original draft, Writing – review & editing. LA-M: Data curation, Formal analysis, Methodology, Resources, Writing – review & editing. BA-K: Conceptualization, Data curation, Methodology, Writing – review & editing. AJ: Writing – original draft, Writing – review & editing.
